# Active–Passive Vibration Control of Cantilever Beam Based on Magnetic Spring with Negative Stiffness and Piezoelectric Actuator

**DOI:** 10.3390/mi16121307

**Published:** 2025-11-21

**Authors:** Min Wang, Zhiwei Jiang, Wei Jiang, Xianghui Feng, Jiheng Ding, Yi Sun, Huayan Pu, Songquan Liao

**Affiliations:** 1 School of Mechatronic Engineering and Automation, Shanghai University, Shanghai 200444, China; jiangzhiwei0202@shu.edu.cn (Z.J.); ding_jiheng@shu.edu.cn (J.D.); yisun@shu.edu.cn (Y.S.); phygood_2001@shu.edu.cn (H.P.); 2 Shanghai Collaborative Innovation Center of Intelligent Sensing Chip Technology, Shanghai University, Shanghai 200444, China; 3 Shangda General Intelligent Robotics Research Institute, Baoshan District, Shanghai 200444, China; 4 Beijing Xiaomi Mobile Software Co., Ltd., Beijing 100085, China; jiangwei10@xiaomi.com (W.J.); fengxianghui@xiaomi.com (X.F.); 5 State Key Laboratory of Mechanical Transmission, Chongqing University, Chongqing 400044, China

**Keywords:** vibration suppression of cantilever beam, magnetic spring with negative stiffness, parallel of positive and negative stiffness, macro-fiber composite, skyhook damping control law

## Abstract

To enhance the low-frequency vibration suppression capability of cantilever beams, a magnetically tunable piezoelectric cantilever beam structure (MTPCBS) is proposed in this paper. A magnetic spring with negative stiffness (NSMS) is fixed at the free end of a cantilever beam, forming a quasi-zero-stiffness structure. Meanwhile, a macro-fiber composite (MFC) patch is bonded near the root of the beam to implement active skyhook damping control for active vibration control. A theoretical model of the cantilever beam, NSMS, and MFC is established, and the displacement transmissibility of the MTPCBS is derived. The influences of the magnet distance of the NSMS and the control gain of the controller are investigated via simulation. Experimental results indicate that compared to the single beam, the effective vibration isolation frequency of the proposed MTPCBS shifts from 15.3 Hz to 4.6 Hz. When subjected to random vibration excitation ranging from 1 to 80 Hz, the root mean square (RMS) value of vibration decreases from 0.03 g to 1.77 × 10^−3^ g, with the vibration attenuation rate improving from −50% to 91%. The proposed MTPCBS and active–passive vibration control method for cantilever beams significantly enhances low-frequency vibration suppression capabilities, providing a feasible strategy for achieving broadband vibration suppression.

## 1. Introduction

Cantilever beams are widely employed in sensing, actuation, energy harvesting, and microelectromechanical systems due to their excellent flexibility and significant free-end displacement [1,2,3,4,5]. As one of the most fundamental structural elements, they are found in high-end equipment such as solar sails, robotic arms, industrial robots, and atomic force microscopes [6,7,8,9]. However, the inherently low stiffness of cantilever beam structures renders them highly sensitive to external excitation and disturbances, making them prone to sustained vibration. This significantly compromises the operational accuracy of high-precision instruments and, in critical cases, leads to fatigue damage, shortening the service life of the equipment [10,11].

Traditional passive vibration control techniques applied to beams primarily focus on the optimization of the beam structure itself or adding substructures to the beam [12]. The former approach alters the natural frequency of the beam by designing the cross-section shape of the beam or using composite materials to avoid common excitation frequency ranges. For example, Nisamudhin K.M. investigated the vibration and damping characteristics of beams with different honeycomb structures [13], and Li researched a rotating three-dimensional cantilever beam with variable cross-section [14]. The latter approach involves attaching a spring–mass–damper dynamic vibration absorber to the beam [15,16,17], which dissipates the vibration energy of the main structure by tuning its frequency to the resonance frequency of the main structure. Qian [18] investigated the exact nonlinear dynamics of a simply supported beam with a nonlinear spring-inerter-damper vibration absorber. Nonlinear energy sinks have also been employed for vibration control of beams [19,20,21]. Alternatively, viscoelastic materials with a high damping factor can be applied to the beam surface to increase structural damping, thereby converting mechanical energy into heat for dissipation [22,23,24,25]. Passive vibration control methods offer significant advantages such as simple principles, high reliability, no external energy requirements, and easy maintenance, but they still have certain limitations [26]. Firstly, the vibration isolation bandwidth is limited. Typically, structural design is optimized for a specific frequency or a narrow frequency bandwidth. Once the external excitation frequency changes, the control effect will decline [27]. Secondly, additional substructures introduce extra mass, increasing structural complexity and rendering them unsuitable for applications requiring weight reduction or encountering low-frequency vibration [28].

Active vibration control aims to address low-frequency vibration [29]. It employs external actuators to apply a controllable force, opposite to the vibration, in real time to suppress vibration. Macro-fiber composite (MFC) exhibits outstanding toughness and driving capability, making it widely used in flexible structures [30]. Raza [31] employed MFC for open-loop active control of a cantilever beam made of composite materials. Other researchers have applied various control algorithms for closed-loop vibration control, including positive position feedback control [32,33], optimal control algorithm [34], adaptive RLS algorithm [35], Fx-LMS algorithm [36,37], fuzzy logic control algorithm [38], and other intelligent algorithms. Zhang [39] and Ma [40] have studied the optimization of the position of MFCs on the surface of beam structures. Qiu [41,42] applied fuzzy neural networks and reinforcement learning to the vibration control of three flexible coupled beams. Although the aforementioned control methods can achieve satisfactory control performance, they are confronted with challenges such as complex controller structures, large computational burden, and challenges to debug and optimize [43].

Since the first-order vibration mode of the beam is the easiest to be excited and the vibration energy is relatively high at low frequencies, in order to achieve broadband vibration suppression of the cantilever beam under low-frequency vibration excitation, a magnetically tunable piezoelectric cantilever beam structure (MTPCBS) is designed, and a hybrid active–passive vibration control method is proposed in this paper. A magnetic spring with negative stiffness (NSMS) composed of multiple magnets, which owns the advantage of being non-contact, is fixed at the free end of the beam and connected in parallel with the beam. Compared to traditional configurations employing three coaxially aligned magnets or annular magnets [44,45,46], the proposed NSMS exhibits symmetry also in the horizontal direction, thereby enhancing spatial symmetry. This design offers additional tunable parameters in the form of adjustable horizontal distance, providing greater flexibility. By symmetrically adjusting either the horizontal or vertical distance, different magnetic force–displacement curves can be achieved, enabling adaptation to various positive stiffness characteristics. A QZS structure based on a parallel of positive and negative stiffness is formed to broaden the vibration isolation bandwidth. Meanwhile, an MFC patch is bonded near the root of the beam. MFC is not solely used for active control but forms a unique hybrid control system with the NSMS. Through the application of the skyhook damping control law, which is straightforward in principle and can be implemented using an analog circuit board alone, the system suppresses resonance around the shifted natural frequency induced by passive control. This approach effectively combines the benefits of both active and passive vibration control. As a result, the resonance peak near the beam’s first natural frequency is markedly attenuated, leading to a significant improvement in low-frequency vibration suppression performance.

This paper is divided into five sections. In Section 2, the dynamic model of the MTPCBS is established, including the modeling of the magnetic force of the NSMS, the modeling of the MFC actuator, and the derivation of the displacement transmissibility. In Section 3, numerical simulations based on the established theoretical model are conducted. The effects of the magnet distance of the NSMS on both the magnetic force and the structural transmissibility are analyzed, and the control performance under different control gains is evaluated. In Section 4, the effectiveness of the proposed hybrid active–passive vibration control method for the cantilever beam is experimentally validated through random, sinusoidal, and frequency-sweep experiments. Finally, the conclusions are summarized in Section 5.

## 2. Structural Description and Modeling of MTPCBS

### 2.1. Structural Description

**Figure 1:**
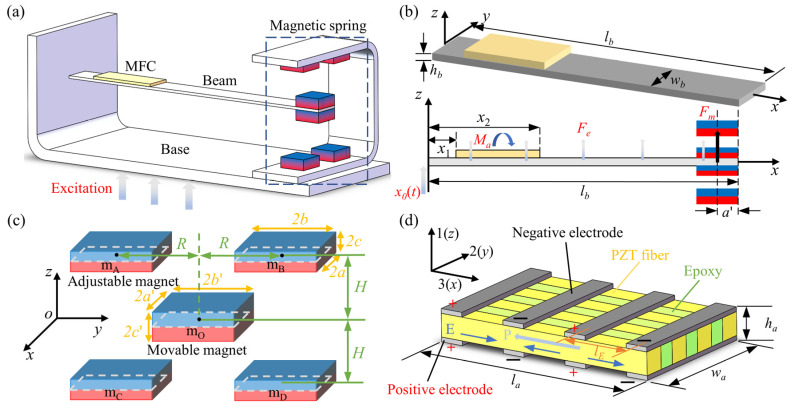
Structural description of the MTPCBS (**a**) Schematic of MTPCBS; (**b**) Geometric parameters of the MTPCBS; (**c**) Geometric parameters of the NSMS; (**d**) Structural composition and geometric parameters of the MFC.

Figure 1 illustrates the structure of the MTPCBS, designed for investigating hybrid active–passive vibration control of the cantilever structure.

As shown in Figure 1a, the cantilever beam is fixed to the base at one end. An MFC is attached to the root of the beam, and a magnetic spring (NSMS) composed of multiple magnets is installed at the free end of the beam. Figure 1b illustrates the definition of the geometric parameters of the beam. A displacement excitation *x*_0_(*t*) is applied at the root of the beam, which is equivalent to imposing a distributed inertial force *F_e_* on the beam. The vibrational response at the free end is then observed. The NSMS acts as a negative stiffness mechanism, counteracting the positive stiffness of the beam. The resulting magnetic force *F_m_* reduces the natural frequency of the MTPCBS, thereby broadening the isolation bandwidth as a passive vibration control method. Simultaneously, an MFC actuator is incorporated, with its starting and ending positions defined as *x*_1_ and *x*_2_, respectively. By integrating an active control algorithm, an active moment *M_a_* is applied to the beam, thereby achieving active vibration control of the beam. Figure 1c defines the geometric parameters of the NSMS. The NSMS consists of four adjustable magnets and one movable magnet, arranged symmetrically in a mutually attractive configuration. The magnitude of both the magnetic force and the negative stiffness can be modulated by adjusting the horizontal distance *R* and the vertical distance *H*. Figure 1d shows the structural composition and dimensional parameters of the P1-type MFC, primarily consisting of two electrode layers, and one active layer made of PZT fiber and epoxy. The specific parameter values of the cantilever beam, NSMS, and MFC are shown in Table 1.

### 2.2. Dynamic Modeling of the MTPCBS

The theoretical dynamic model of the MTPCBS is established in this part. When a displacement excitation *x*_0_(*t*) is applied at the fixed end of the beam, the displacement at point *x* along the beam at time *t* can be expressed as follows:

(1) $$z^{*}(x,t)=x_{0}(t)+z(x,t)$$

where *z*(*x*,*t*) represents the elastic vibration of the beam caused by external excitation.

According to Euler–Bernoulli beam theory, the differential equation for the lateral elastic vibration of the beam is [37,47,48,49] as follows:

(2) $$EI\frac{\partial^{4}z(x,t)}{\partial x^{4}}+\rho A\frac{\partial^{2}z(x,t)}{\partial t^{2}}=F(x,t)+\frac{\partial^{2}M_{a}(x,t)}{\partial x^{2}}$$

where *E* denotes the Young’s modulus of the beam and *I* represents the area moment of inertia of the cross-section. *ρ* and *A* represent the density and cross-sectional area of the beam, respectively. *F*(*x*,*t*) and *M_a_*(*x*,*t*) represent the external force and external moment acting on the beam, respectively.

Using the modal superposition method, the solution to the equation is

(3) $$z(x,t)=\sum_{i=1}^{\infty }\varphi_{i}(x)q_{i}(t)$$

where $\varphi_{i}(x)$ and $q_{i}(t)$ denote the ith-order mode shape function and generalized coordinate. Ignoring the influence of MFC and NSMS on the stiffness and mass of the beam, the mode shape function and frequency equation under the boundary conditions of the cantilever beam can be expressed as follow:

(4) $$\varphi_{i}(x)=\cosh\left(\beta_{i}x\right)-\cos\left(\beta_{i}x\right)-\frac{\cosh\left(\beta_{i}l_{b}\right)+\cos\left(\beta_{i}l_{b}\right)}{\sinh\left(\beta_{i}l_{b}\right)+\sin\left(\beta_{i}l_{b}\right)}\left(\sinh\left(\beta_{i}x\right)-\sin\left(\beta_{i}x\right)\right)$$

(5) $$\cos\left(\beta_{i}l_{b}\right)\cosh\left(\beta_{i}l_{b}\right)=-1$$

Substitute Equation (3) into Equation (2), multiply the equation of motion by the jth-order mode shape function $\varphi_{j}(x)$, and perform an integral operation along the beam length *l_b_* to obtain

(6) $$\sum_{i=1}^{\infty }q_{i}(t)\int_{0}^{l_{b}}EI\varphi_{j}(x)\varphi_{i}^{\left(4\right)}(x)dx+\sum_{i=1}^{\infty }\overset{\ldots }{q_{i}}(t)\cdot \rho A\int_{0}^{l_{b}}\varphi_{j}(x)\varphi_{i}(x)dx=\int_{0}^{l_{b}}\left[F(x,t)+\frac{\partial^{2}M_{a}(x,t)}{\partial x^{2}}\right]\varphi_{j}(x)dx$$

By utilizing the orthogonality property of the natural mode shapes, the following expression is further derived:

(7) $$\overset{\cdot \cdot }{q_{i}}(t)+{\omega_{i}}^{2}q_{i}(t)=\frac{1}{M_{i}}\int_{0}^{l_{b}}F(x,t)\varphi_{i}(x)dx+\frac{1}{M_{i}}\int_{0}^{l_{b}}\frac{\partial^{2}M_{a}(x,t)}{\partial x^{2}}\varphi_{i}(x)dx$$

Next, the expressions for the external force and external moment acting on the beam are derived. The external force *F*(*x*,*t*) comprises the inertial force *F_e_* induced by the distributed mass of the beam under excitation *x*_0_(*t*), and the magnetic force *F_m_* provided by the NSMS. The inertial force *F_e_* can be expressed as follows:

(8) $$F_{e}(x,t)=-\rho A\cdot \overset{\cdot \cdot }{x_{0}}(t)$$

### 2.3. Modeling of NSMS

As shown in Figure 1c, the NSMS consists of four adjustable magnets and one movable magnet, arranged symmetrically in a mutually attractive configuration. The geometric dimensions of two kinds of magnets are specified as 2*a* × 2*b* × 2*c* and 2*a*′ × 2*b*′ × 2*c*′, respectively. A coordinate system is established at the geometric center of the movable magnet M_O_ when it is at the equilibrium position. The coordinates of the adjustable magnets are defined as $\left(X,Y,Z\right)$. The horizontal distance *R* and vertical distance *H* represent the distance between the geometric centers of the magnets. At time *t*, when the distance of the movable magnet M_O_ from the equilibrium position is $z\left(l_{b}-a\prime ,t\right)$, M_O_ experiences a resultant force in the Z-direction due to the combined interaction with the four magnets: M_A_ (0, -*R*, *H*), M_B_ (0, *R*, *H*), M_C_ (0, -*R*, -*H*), M_D_ (0, *R*, -*H*). Based on the magnetic-charge model originally proposed by Akoun and Yonnet and subsequently validated in multiple studies [50,51,52], the magnetic force *F_m_* can be obtained by the following formula:

(9) $$F_{m}=F_{z}(0,R,z-H)+F_{z}(0,-R,z-H)+F_{z}(0,R,z+H)+F_{z}(0,-R,z+H)$$

with

(10) $$F_{z}=\frac{JJ^{\prime }}{4\pi \mu_{0}}\sum_{i=0}^{1}\sum_{j=0}^{1}\sum_{k=0}^{1}\sum_{l=0}^{1}\sum_{p=0}^{1}\sum_{q=0}^{1}{\left(-1\right)}^{i+j+k+l+p+q}\Phi_{z}\left(U_{ij},V_{kl},W_{pq},r\right)\;\;\Phi_{z}(U,V,W,r)=-UW\ln(r-U)-VW\ln(r-V)+UV\arctan\left(\frac{UV}{rW}\right)-rW$$

where vacuum permeability $\mu_{0}=4\pi \times 10^{-7}$ and the variables *U*, *V*, *W*, and *r* are listed as follows:

(11) $$U_{ij}\;=x\prime +{\left(-1\right)}^{j}a\prime -{\left(-1\right)}^{i}a,\;\;V_{kl}\;=y\prime +{\left(-1\right)}^{l}b\prime -{\left(-1\right)}^{k}b,\;\;W_{pq}=z\prime +{\left(-1\right)}^{q}c\prime -{\left(-1\right)}^{p}c,\;\;r=\sqrt{U_{ij}^{2}+V_{kl}^{2}+W_{pq}^{2}}$$

where (x′,y′,z′) represents the position of the movable magnet relative to the adjustable magnet, as given in Equation (9).

To simplify subsequent calculations, a fifth-order polynomial is used to fit the magnetic force of the NSMS. The simplified magnetic force acting on the beam is obtained as follows:

(12) $$\begin{array}{ll} F_{m}(x,t) & =[P_{1}\cdot z\left(l_{b}-a\prime ,t\right)+P_{3}\cdot z{\left(l_{b}-a\prime ,t\right)}^{3}+P_{5}\cdot z{\left(l_{b}-a\prime ,t\right)}^{5}]\cdot \delta \left(x-l_{b}+a\prime \right)\\ & =F_{m}(t)\cdot \delta \left(x-l_{b}+a\prime \right) \end{array}$$

where *P*_1_, *P*_3_, and *P*_5_ represent the coefficients of the first-order, third-order, and fifth-order terms. δ(x) is the Dirac function.

**Figure 2:**
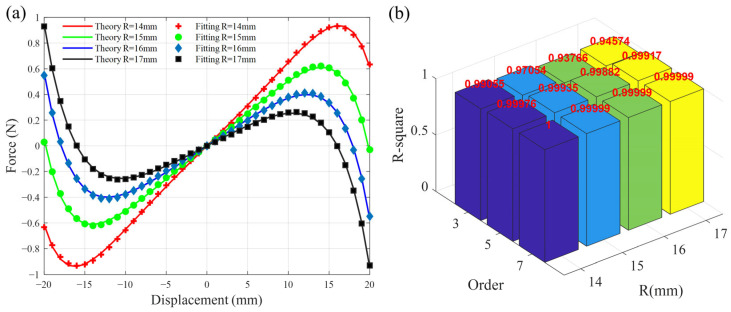
(**a**) Theoretical and fitting force-displacement curves with different horizontal distances *R*; (**b**) R-square for the approximated forces with varying *R* and order.

To validate the fitting accuracy, Figure 2a presents a comparative plot of the theoretical curves and their corresponding fifth-order polynomial fits with various horizontal distances *R*. The R-squared is utilized to assess the degree of fitting error. Figure 2b presents the R-square for the approximated forces with varying *R* and order, compared to their respective theoretical values. Both the fifth-order and seventh-order degree polynomials exhibit excellent fitting degrees, consistently exceeding 0.99. Therefore, considering the balance between the accuracy of fitting and the complexity of solving the dynamic model, it is reasonable to approximate nonlinear magnetic forces with a fifth-order polynomial.

### 2.4. Modeling of MFC

A P1-type MFC is employed as the actuator, as illustrated in Figure 1d, to apply a moment *M_a_*(*x*,*t*) to the beam for active vibration control. The relationship between the stress $\sigma_{a}$ generated by MFC and the input voltage $V_{a}(t)$ can be expressed as follows:

(13) $$\sigma_{a}=E_{a}\cdot \frac{d_{33}}{l_{E}}V_{a}(t)$$

where *E_a_* denotes the elastic modulus of the MFC, *d*_33_ represents the piezoelectric constant, and *l_E_* indicates the distance between adjacent positive electrodes and negative electrodes.

The resulting bending moment is as follows [37,53]:

(14) $$\begin{array}{ll} M_{a}(x,t) & =\int_{\frac{1}{2}h_{b}}^{\frac{1}{2}h_{b}+h_{a}}\sigma_{a}\cdot w_{a}\cdot zdz\cdot \left[H\left(x-x_{1}\right)-H\left(x-x_{2}\right)\right]\\ & =\frac{1}{2l_{E}}h_{a}\left(h_{a}+h_{b}\right)E_{a}\cdot d_{33}\cdot w_{a}\cdot V_{a}(t)\cdot \Delta H(x)=\theta_{a}\cdot \Delta H(x)\cdot V_{a}(t) \end{array}$$

where $H\left(x\right)$ is the Heaviside step function.

Substituting Equations (8), (12) and (14) into Equation (7), and considering the structural and material damping effects, the equation of motion of the MTPCBS can be expressed as follows:

(15) $${\ddot{q}}_{i}(t)+2\xi_{i}\omega_{i}{\dot{q}}_{i}(t)+{\omega_{i}}^{2}q_{i}(t)=\frac{\theta_{a}}{M_{i}}\cdot \left[\varphi_{i}^{\prime }\left(x_{2}\right)-\varphi_{i}^{\prime }\left(x_{1}\right)\right]\cdot V_{a}(t)+\frac{\varphi_{i}(l_{b}-a\prime )}{M_{i}}\cdot F_{m}(t)-\frac{\rho A}{M_{i}}\int_{0}^{l_{b}}\varphi_{i}(x)dx\cdot \overset{\cdot \cdot }{x_{0}}(t)$$

where $\xi_{i}$ represents the damping ratio corresponding to the ith-order mode.

### 2.5. State Space Representation

Introducing the state variable $x(t)={\left[\begin{array}{ll} q_{n}(t) & {\dot{q}}_{n}(t) \end{array}\right]}^{T}$, the equation of motion of the MTPCBS is expressed in state-space form as follows:

(16) $$\begin{array}{l} \dot{x}(t)=Ax(t)+Bu(t)\\ y(t)=Cx(t) \end{array}$$

where **A**, **B**, and **C** denote the state matrix, input matrix, and output matrix, respectively. **u**(*t*) and **y**(*t*) represent the input vector and output vector, respectively, with their specific forms given as follows:

$$A={\left[\begin{array}{cc} 0_{n\times n} & I_{n\times n}\\ -\Omega & -2\Delta \end{array}\right]}_{2n\times 2n}\;\Omega =\mathrm{diag}[{\omega_{1}}^{2},{\omega_{2}}^{2},\ldots ,{\omega_{n}}^{2}]\;\Delta =\mathrm{diag}[\xi_{1}\omega_{1},\xi_{2}\omega_{2},\ldots ,\xi_{n}\omega_{n}]\;\;B={\left[\begin{array}{ccc} 0_{n\times 1} & 0_{n\times 1} & 0_{n\times 1}\\ \frac{\theta_{a}\left[\varphi_{n}^{\prime }\left(x_{2}\right)-\varphi_{n}^{\prime }\left(x_{1}\right)\right]}{M_{i}} & \frac{\varphi_{n}\left(l_{b}-a^{\prime }\right)}{M_{i}} & \frac{-\rho A\int_{0}^{l_{b}}\varphi_{n}(x)dx}{M_{i}} \end{array}\right]}_{2n\times 3}\;\;u(t)={\left[\begin{array}{l} V_{a}(t)\\ F_{m}(t)\\ \overset{\cdot \cdot }{x_{0}}(t) \end{array}\right]}_{3\times 1}\;C={\left[\begin{array}{cc} I_{n\times n} & 0_{n\times n} \end{array}\right]}_{n\times 2n}\;y(t)={\left[q_{n}(t)\right]}_{n\times 1}$$

### 2.6. Displacement Transmissibility

The displacement response at the free end of the cantilever beam is

(17) $$z_{tip}^{*}(t)=x_{0}(t)+\sum_{i=1}^{n}\varphi_{i}(l_{b})q_{i}(t)$$

The vibration suppression performance is evaluated by calculating the displacement transmissibility as follows:

(18) $$T=20\cdot \log\frac{z_{tip}^{*}(t)}{x_{0}(t)}=20\cdot \log(1+\frac{\sum_{i=1}^{n}\varphi_{i}(l_{b})q_{i}(t)}{x_{0}(t)})$$

### 2.7. Control Strategy

Skyhook damping control is an effective approach for mitigating resonance at the natural frequency. Accordingly, an integral-acceleration feedback (IAF) strategy is employed in the controller design to suppress the resonance at the first natural frequency of the beam. The relationship between the control voltage and the feedback acceleration signal can be expressed as follows:

(19) $$V_{a}(t)=k\cdot \frac{1}{s}\cdot \overset{\cdot \cdot }{z_{tip}^{*}}(t)$$

where *k* denotes the integral gain coefficient, and 1/s represents the integrator term in the Laplace domain.

## 3. Numerical Simulations

### 3.1. Simulation Model

**Figure 3:**
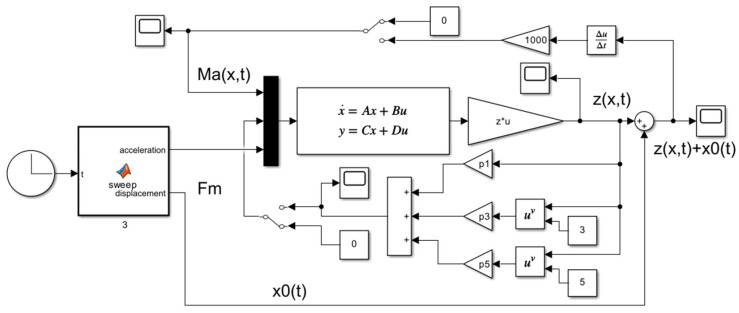
The simulation model in Simulink.

**Figure 4:**
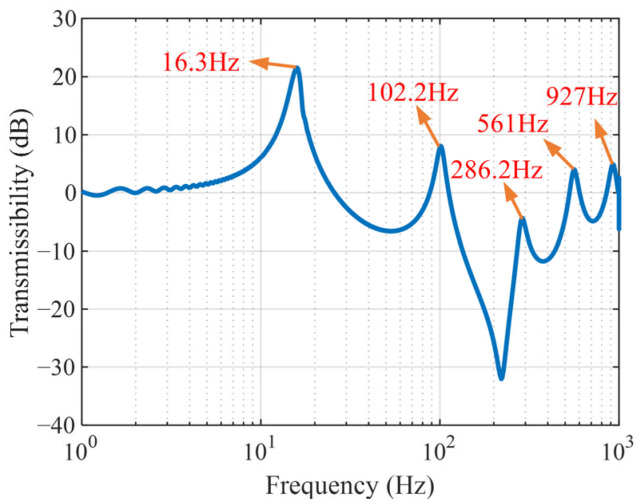
The displacement transmissibility curve of the single cantilever beam.

According to the theoretical formula in Section 2, a simulation model is built in MATLAB R2023a, as shown in Figure 3. The first five natural frequencies of the single cantilever beam, corresponding to the parameters provided in Table 1, are calculated, and the displacement transmissibility is plotted, as illustrated in Figure 4. Since the first-order mode is most susceptible to excitation under broad-frequency excitation and its vibrational energy typically dominates the overall response, this study focuses on vibration suppression with respect to the first-order mode of the beam.

### 3.2. Effect of Passive Control

**Figure 5:**
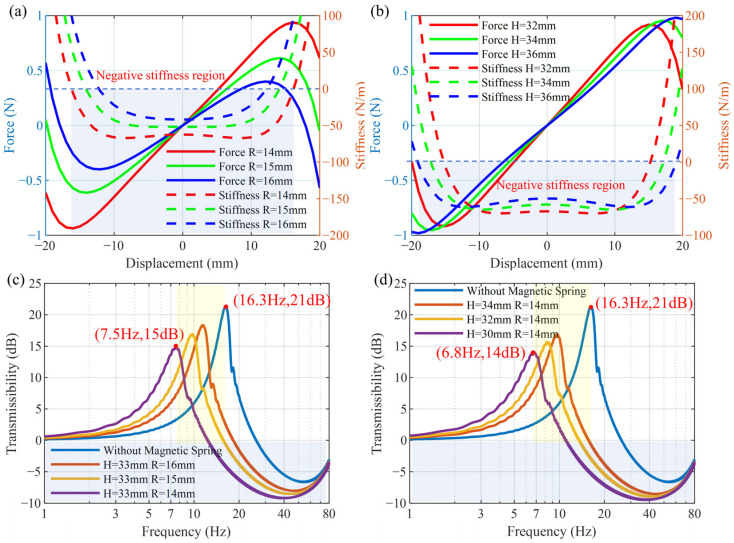
Effect of NSMS on beam vibration: (**a**,**b**) the magnetic force and stiffness of the NSMS with respect to (**a**) horizontal distance *R* when *H* = 33 mm, (**b**) vertical distance *H* when *R* = 14 mm; (**c**,**d**) displacement transmissibility with respect to (**c**) horizontal distance *R* (**d**) vertical distance *H*.

The NSMS, composed of multiple magnets with adjustable distance, is coupled with the beam as a negative stiffness mechanism, thereby forming a parallel structure with positive and negative stiffness. Based on the model of the NSMS presented in Section 2.3, the variations of magnetic force and stiffness generated by the NSMS with respect to magnet distance are discussed, as illustrated in Figure 5a,b. When the vertical distance *H* = 33 mm and the horizontal distance *R* increases from 14 mm to 16 mm, the NSMS exhibits negative stiffness characteristics over a certain displacement range. Moreover, as the horizontal distance *R* increases, both the magnetic force and stiffness show a decreasing trend.

When the horizontal distance *R* is fixed at 14 mm and the vertical distance *H* increases from 32 mm to 36 mm, both the magnetic force and stiffness also demonstrate a decreasing trend. However, compared with changing the parameter *R*, the variation is relatively small. Figure 5c,d demonstrate the influence of horizontal distance and vertical distance on the displacement transmissibility, respectively. Without the NSMS, the first natural frequency of the beam is 16.3 Hz, and the resonance peak is 21 dB. As the horizontal distance *R* of the NSMS decreases, the natural frequency shifts forward to 7.5 Hz, and the peak amplitude is reduced to 15 dB. Similarly, as the vertical distance *H* decreases, the natural frequency shifts forward to 6.8 Hz, and the peak amplitude is reduced to 14 dB.

### 3.3. Effect of Active Control

**Figure 6:**
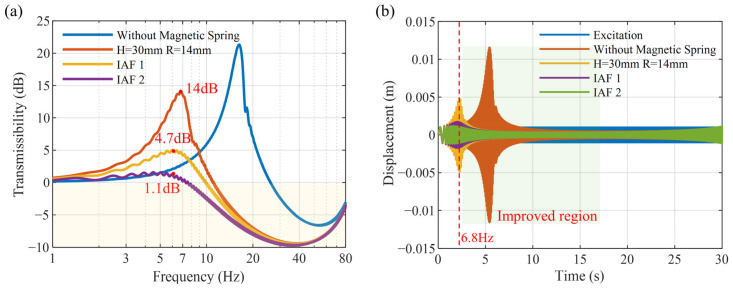
Active vibration control performance in (**a**) the frequency domain and (**b**) the time domain.

When *H* = 30 mm and *R* = 14 mm, active vibration control simulations are carried out. The control results based on the IAF law are presented in Figure 6. As shown in Figure 6a, with the increase in control gain, the amplitude at the natural frequency of 6.8 Hz gradually decreases from 14 dB to 4.7 dB and then to 1.1 dB, thereby effectively mitigating the resonance near the first natural frequency. Figure 6b presents a comparison of the time-domain response curves under a linear frequency-sweep excitation ranging from 1 to 90 Hz, with a duration of 30 s and a constant displacement amplitude of 1 mm. The natural frequency of the MTPCBS is moved forward to 6.8 Hz by using the NSMS, significantly suppressing the resonance originally observed around 16.3 Hz, but at the same time, the low-frequency vibration is amplified. The implementation of the skyhook damping control method reduces the resonance peak in the low-frequency region, maintaining the low-frequency transmissibility nearly identical to that of the original beam without the NSMS.

## 4. Experimental Verification

### 4.1. Experimental Setup

**Figure 7:**
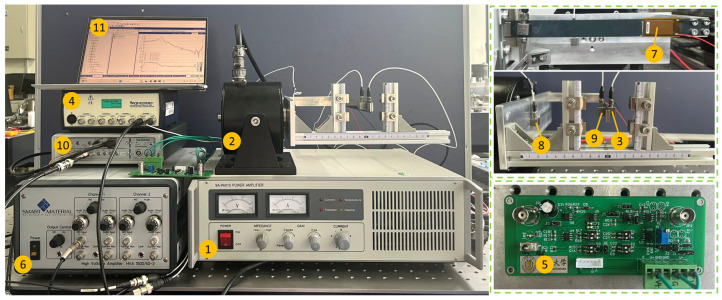
Experimental setup: (1) power amplifier SA-PA010; (2) exciter SA-JZ005; (3) feedback accelerometer PCB333B52; (4) sensor signal conditioner; (5) controller; (6) high-voltage amplifier HVA 1500/50-2; (7) P1-type MFC; (8) and (9) accelerometer; (10) LMS SCADAS mobile SCM2E01; (11) computer.

The experimental setup is shown in Figure 7. The base platform of the MTPCBS was rigidly connected to the output shaft of the shaker. Two accelerometers (PCB333B52, PCB Piezotronics, Inc., Depew, NY, USA) were used to measure the vibration transmissibility between the base platform and the beam. A data acquisition instrument (LMS SCADAS mobile SCM2E01, Siemens Digital Industries Software, Plano, TX, USA) was employed to collect acceleration signals, and the data were processed and analyzed via the software LMS Test. Lab 18. The LMS SCADAS mobile also outputs a voltage signal, amplified by a power amplifier (SA-PA010, Wuxi Shiao Technology Co., Ltd., Wuxi, China) to drive the shaker (SA-JZ005, Wuxi Shiao Technology Co., Ltd., Wuxi, China) to generate excitation. The feedback signal, acquired by the feedback sensor (PCB333B52), was conditioned by a signal conditioner before being fed into the controller. The controller was implemented using an analog circuit board, and its output signal was amplified with a high-voltage amplifier (HVA 1500/50-2, Smart Material Corp., Sarasota, FL, USA) to drive the MFC.

### 4.2. Verification of the Parallel of Positive and Negative Stiffness

**Figure 8:**
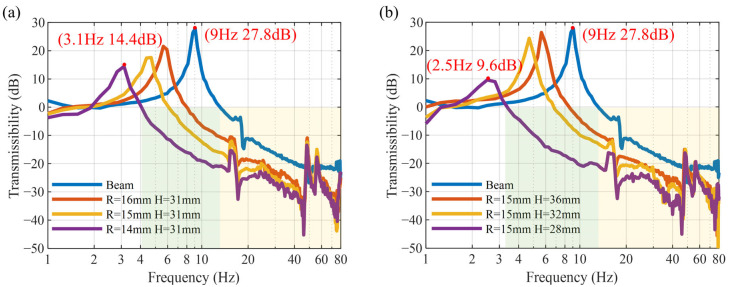
Acceleration transmissibility with respect to (**a**) horizontal distance *R* and (**b**) vertical distance *H*.

The influence of parameters of NSMS on the vibration of the MTPCBS is verified, including the horizontal distance *R* and the vertical distance *H*. As shown in Figure 8a, without the NSMS (blue curve), the first-order natural frequency of the beam is 9 Hz, and the corresponding resonance peak is 27.8 dB. With NSMS, while maintaining a constant vertical distance *H* = 31 mm, the natural frequency gradually decreased and the resonance peak reduced as the horizontal distance *R* decreased from 16 mm to 14 mm, indicating an increase in negative stiffness. When *R* = 14 mm, the natural frequency reached 3.1 Hz with a resonance peak of 14.4 dB. When the horizontal distance *R* = 15 mm, as the vertical distance *H* decreases from 36 mm to 28 mm, the acceleration transmissibility curve exhibits a similar trend: The natural frequency shifts forward and the resonance peak decreases. When *R* = 15 mm and *H* = 28 mm, the negative stiffness provided by the NSMS counterbalanced the positive stiffness of the beam, resulting in a quasi-zero-stiffness characteristic. The MTPCBS achieved a natural frequency of 2.5 Hz and a resonance peak of 9.6 dB, significantly broadening the vibration isolation bandwidth.

### 4.3. Verification of Active Vibration Control

**Figure 9:**
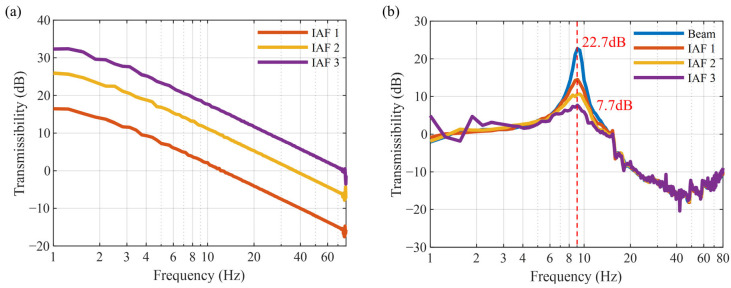
Active vibration control performance with different control gains: (**a**) transmissibility of the controller; (**b**) transmissibility of MTPCBS.

**Figure 10:**
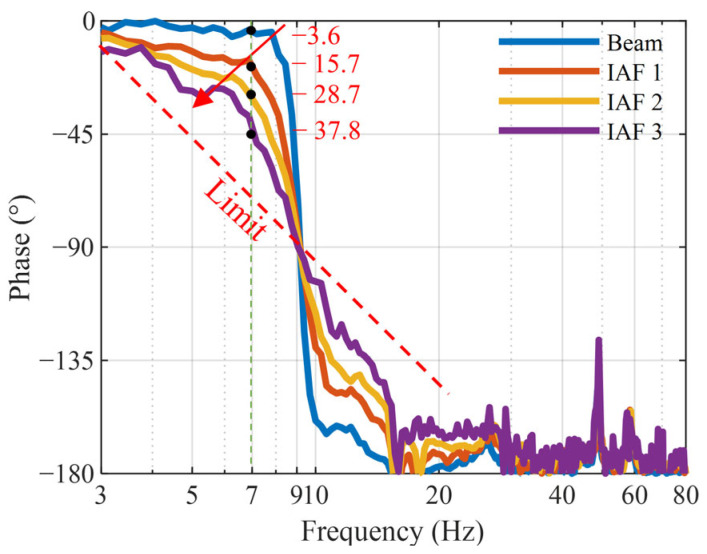
Phase angle curves with different control gains.

This section validates the control performance of the IAF control law. Since the controller is implemented using an analog circuit board, the adjustment of the control gain is achieved through a potentiometer, making it difficult to quantify the values of the control gain precisely. Therefore, three different control gain levels are selected and labeled as IAF 1 to IAF 3, and the corresponding controller transfer functions are shown in Figure 9a. As demonstrated in Figure 9b, the amplitude of the transmissibility at the resonance peak decreases from 22.7 dB to 7.7 dB as the control gain increases. Figure 10 presents the phase–frequency diagrams corresponding to different control gains. Stability analysis constitutes an essential component of control system design. After the incorporation of active control, the system maintains relative stability. As the gain factor increases (from IAF 1 to IAF 3), the peak magnitude of the system becomes suppressed, while the phase angle exhibits continuous growth. This indicates that although higher gain factors can enhance the vibration isolation performance of the system, they also contribute to increased time delay. The time delay in the mechanical transmission link is merely −3.6 degrees (at 7 Hz) under passive conditions, which is insufficient to induce instability. However, with the introduction of the feedback control, when the control gain is increased to IAF 3, the phase delays at 7 Hz reach −15.7 degrees, −28.7 degrees, and −37.8 degrees, respectively. It can be clearly observed that although the phase delay continues to increase, it remains considerably distant from the critical limit. Within the gain range from IAF 1 to IAF 3, if the system were to become unstable, the phase curve would exhibit chaotic characteristics, and the vibration isolation performance would deteriorate. Experimental observations confirm that the phase maintains regular variations and remains at a safe distance from the instability threshold, demonstrating that the system remains stable within this gain range. Nevertheless, it is worth noting that excessively high gains may pose potential instability risks, and the effectiveness of active control is limited.

### 4.4. Comparison of Vibration Isolation Performance

**Figure 11:**
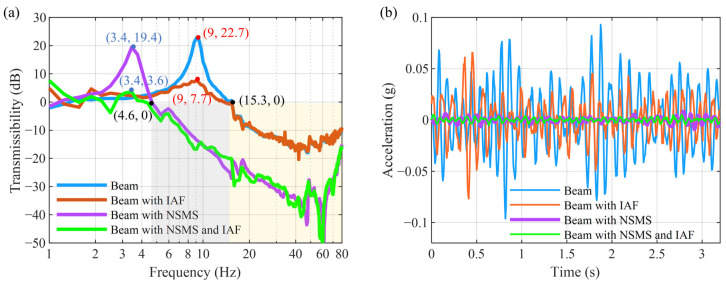
Comparison of vibration isolation performance: (**a**) transmissibility; (**b**) time-domain curve.

Figure 11a,b present the comparison of vibration isolation performance in the frequency domain and the time domain, respectively.

The applied excitation consists of random vibration excitation ranging from 1 to 80 Hz. The NSMS makes the natural frequency of the beam reduce from 9 Hz to 3.4 Hz and shifts the initial vibration isolation frequency from 15.3 Hz to 4.6 Hz, thereby improving the isolation performance within the range of 4.6 Hz to 80 Hz. However, vibration amplification occurred near the new natural frequency of 3.4 Hz, increasing from approximately 0 dB to 19.4 dB. The single IAF control algorithm can reduce the resonance peak from 22.7 dB to 7.7 dB. The combination of the NSMS and the IAF control law maintains the transmissibility near the natural frequency of 3.4 Hz, identical to that of the original single beam. The vibration isolation effect can also be intuitively reflected through the time-domain curve in Figure 11b. When the MTPCBS is subjected to a random excitation with a root mean square (RMS) amplitude of 0.02 g, the response RMS values for the beam alone, beam with IAF, beam with NSMS, and beam with NSMS and IAF are 0.03 g, 0.02 g, 2.6 × 10^−3^ g, and 1.77 × 10^−3^ g, respectively. Detailed data are compared in Table 2.

**Figure 12:**
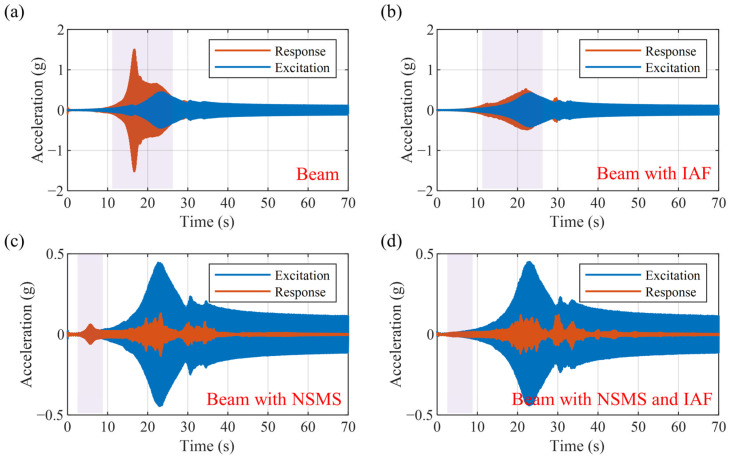
Comparison of time-domain sweep frequency experimental curves: (**a**) beam; (**b**) beam with IAF; (**c**) beam with NSMS; (**d**) beam with NSMS and IAF.

Figure 12 shows a time-domain comparison of the above four cases under a linear sweep excitation from 1 to 35 Hz over a duration of 70 s. The blue curve represents the input excitation, while the orange curve denotes the response. In Figure 12a, the vibration of the beam around 9 Hz is significantly amplified. In Figure 12b, applying active vibration control effectively suppresses the resonance near 9 Hz. It can be seen from Figure 12c that the NSMS can attenuate the vibration of the beam starting from approximately 5 Hz. In Figure 12d, active control is applied to prevent the vibration at low frequencies from being amplified.

**Figure 13:**
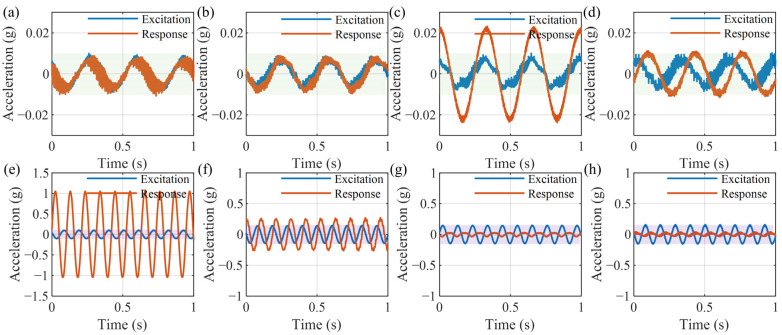
Time-domain response curves under sinusoidal excitation: (**a**–**d**) 3 Hz; (**e**–**h**) 9.5 Hz: (**a**) and (**e**) beam; (**b**) and (**f**) beam with IAF; (**c**) and (**g**) beam with NSMS; (**d**) and (**h**) beam with NSMS and IAF.

Figure 13 shows the time-domain response curves corresponding to the two characteristic excitation frequencies of 3 Hz and 9.5 Hz, respectively. Under a sinusoidal excitation with a frequency of 3 Hz and an amplitude of 0.01 g, the response amplitude of the single beam is 0.01 g (Figure 13a). The active control has no effect on this frequency, so the amplitude remains 0.01 g (Figure 13b). Using only the NSMS will amplify the amplitude to 0.02 g (Figure 13c). After applying active control, the amplitude is maintained near 0.01 g (Figure 13d). Under sinusoidal excitation at 9.5 Hz with an amplitude of 0.1 g, the response amplitude of the single beam is amplified to 1 g (Figure 13e). The application of active control reduces the amplitude to 0.27 g (Figure 13f). The NSMS significantly reduces the amplitude to 0.03 g (Figure 13g). Since the active control does not function at this frequency (9.5 Hz), the amplitude in Figure 13h remains consistent with that in Figure 13g.

## 5. Conclusions

This paper proposes a hybrid active–passive vibration control method for cantilever beams. Multiple magnets are fixed at the free end of the beam in a symmetrically attractive arrangement, forming a magnetic spring that provides negative stiffness. Combined with the beam, a parallel structure with positive and negative stiffness is formed, serving as a passive control method to achieve vibration attenuation. Concurrently, an MFC is bonded near the root of the beam and integrated with a skyhook damping control algorithm to achieve active vibration control.

This paper begins with the theoretical modeling of the MTPCBS, deriving an analytical expression for the displacement transmissibility under transverse vibration. Through simulation analysis, the influence of parameters of NSMS: horizontal distance and vertical distance on the transmissibility, as well as control performance under varying control gains, is examined. Experimental validation is performed using random, frequency-sweep, and sinusoidal excitations, confirming the conclusions drawn from both the theoretical and simulation analyses. The following quantitative conclusions can be drawn:

(a) By adjusting the magnet distance, a QZS structure can be achieved. The natural frequency of the MTPCBS is reduced from 9 Hz to 3.4 Hz, broadening the vibration suppression bandwidth. Under identical excitation conditions, the RMS vibration amplitude decreased from 0.03 g to 2.6 × 10^−3^ g, corresponding to an improvement in vibration attenuation from a 50% amplification to an 87% reduction.
(b) With the implementation of active skyhook damping control, the resonance peak was reduced from 19.4 dB to 3.6 dB, and the RMS amplitude reached 1.77 × 10^−3^ g, achieving a vibration attenuation rate of 91%. The proposed active–passive vibration control method enhances the low-frequency vibration suppression capacity of the beam, offering a viable strategy for attenuating low-frequency vibrations in beam-type structures.

From a purely academic research perspective, hybrid active–passive control undoubtedly holds significant appeal. However, its practical implementation in engineering must be evaluated against a broader set of factors, such as space limitations, energy constraints, and excitation conditions. Passive control is generally more suitable for broadband vibration isolation. In scenarios with limited energy supply or stringent reliability requirements, a purely passive solution may represent the more prudent choice. Conversely, active control is primarily effective at suppressing vibrations around the resonance frequency. Thus, in spatially constrained applications where the dominant excitation coincides with the system’s natural frequency, applying active control alone is the preferred option.

The proposed active–passive vibration control method for cantilever beams based on magnetic springs and piezoelectric actuators has been validated in principle. However, improvements are still needed. First, structural optimization is required in engineering applications, specifically the integrated design of the four adjustable magnets in the NSMS with the beam. Second, the active control performance needs further enhancement. To address the limitations caused by feedback control delays, introducing feedforward control could further improve control effectiveness. In addition, the control effects of the NSMS and MFC on higher-order modes of the beam and wideband excitation also urgently need to be studied.

## Figures and Tables

**Table 1:** Geometric and physical parameters of the MTPCBS.

Parameters		Value	Unit
Cantilever beam	Length (*l_b_*) × width (*w_b_*) × thickness (*h_b_*)	200 × 18 × 0.8	mm^3^
	Elasticity modulus	200	GPa
	Density	7850	kg/m^3^
NSMS	Length (2*a*) × width (2*b*) × height (2*c*) of Adjustable magnet	15 × 15 × 5	mm^3^
	Length (2*a′*) × width (2*b′*) × height (2*c′*) of Movable magnet	15 × 15 × 4	mm^3^
	Polarization *J*	1.42	T
MFC	Length (*l_a_*) × width (*w_a_*) × thickness (*h_a_*)	28 × 14 × 0.3	mm^3^
	Elastic modulus	30.336	GPa
	Piezoelectric constant *d*_33_	374 × 10^−12^	C/N

**Table 2:** Comparison of experimental data in the time and frequency domains.

Parameters	Beam	Beam with IAF	Beam with NSMS	Beam with NSMS and IAF	Unit
Resonance frequency	9	9	3.4	3.4	Hz
Resonance peak	22.7	7.7	19.4	3.6	dB
Initial vibration isolation frequency	15.3	15.3	4.6	4.6	Hz
RMS of vibration amplitude	0.03	0.02	2.6 × 10^−3^	1.77 × 10^−3^	g
Vibration attenuation rate	−50%	0%	87%	91%	-

## Data Availability

The data supporting the findings of this paper are available from the corresponding authors upon request.
